# Transcriptional and translational adaptation to aerobic nitrate anabolism in the denitrifier *Paracoccus denitrificans*

**DOI:** 10.1042/BCJ20170115

**Published:** 2017-05-10

**Authors:** Victor M. Luque-Almagro, Isabel Manso, Matthew J. Sullivan, Gary Rowley, Stuart J. Ferguson, Conrado Moreno-Vivián, David J. Richardson, Andrew J. Gates, M. Dolores Roldán

**Affiliations:** 1 Departamento de Bioquímica y Biología Molecular, Universidad de Córdoba, Edificio Severo Ochoa, 1ª planta, Campus de Rabanales, Córdoba 14071, Spain; 2 School of Biological Sciences, University of East Anglia, Norwich Research Park, Norwich NR4 7TJ, U.K.; 3 Department of Biochemistry, University of Oxford, South Parks Road, Oxford OX1 3QU, U.K.

**Keywords:** global nitrogen regulator Ntr, nitrate assimilation, nitrate reductase, nitrate/nitrite-sensor protein, nitrogen regulation, *Paracoccus*

## Abstract

Transcriptional adaptation to nitrate-dependent anabolism by *Paracoccus denitrificans* PD1222 was studied. A total of 74 genes were induced in cells grown with nitrate as N-source compared with ammonium, including *nasTSABGHC* and *ntrBC* genes. The *nasT* and *nasS* genes were cotranscribed, although *nasT* was more strongly induced by nitrate than *nasS*. The *nasABGHC* genes constituted a transcriptional unit, which is preceded by a non-coding region containing hairpin structures involved in transcription termination. The *nasTS* and *nasABGHC* transcripts were detected at similar levels with nitrate or glutamate as N-source, but *nasABGHC* transcript was undetectable in ammonium-grown cells. The nitrite reductase NasG subunit was detected by two-dimensional polyacrylamide gel electrophoresis in cytoplasmic fractions from nitrate-grown cells, but it was not observed when either ammonium or glutamate was used as the N-source. The *nasT* mutant lacked both *nasABGHC* transcript and nicotinamide adenine dinucleotide (NADH)-dependent nitrate reductase activity. On the contrary, the *nasS* mutant showed similar levels of the *nasABGHC* transcript to the wild-type strain and displayed NasG protein and NADH–nitrate reductase activity with all N-sources tested, except with ammonium. Ammonium repression of *nasABGHC* was dependent on the Ntr system. The *ntrBC* and *ntrYX* genes were expressed at low levels regardless of the nitrogen source supporting growth. Mutational analysis of the *ntrBCYX* genes indicated that while *ntrBC* genes are required for nitrate assimilation, *ntrYX* genes can only partially restore growth on nitrate in the absence of *ntrBC* genes. The existence of a regulation mechanism for nitrate assimilation in *P. denitrificans*, by which nitrate induction operates at both transcriptional and translational levels, is proposed.

## Introduction

The soil denitrifier *Paracoccus denitrificans* PD1222 makes multiple metabolic uses of nitrate: (i) as a respiratory electron acceptor to support anaerobic growth, catalyzed by a membrane-bound nitrate reductase (Nar); (ii) as an electron sink to dispose of excess reductant during aerobic metabolism of highly reduced carbon substrates, catalyzed by the periplasmic nitrate reductase (Nap); and (iii) as the nitrogen source for anabolism during both oxic and anoxic growth. The first two processes have been widely studied [1], but the biochemistry and regulation of the third process, nitrate assimilation, has received much less attention. The nitrate assimilation system (Nas) is encoded by the *nasABGHC* gene cluster, which contains a nitrate transporter (NasA), an nicotinamide adenine dinucleotide (NADH)-dependent nitrite reductase (NasB), a small Rieske-type protein required for both nitrate and nitrite reduction (NasG), a nitrite transporter (NasH), and a nitrate reductase (NasC), which receives electrons from NADH via NasB and NasG [2]. The NasB and NasG proteins are essential for growth with nitrate or nitrite as the sole nitrogen source under both aerobic and anaerobic conditions. However, the NasA and NasC proteins are required for nitrate import and reduction only under aerobic conditions because of a functional overlap with biochemical components of the respiratory nitrate reductase system that are synthesized under anaerobic conditions [2,3].

Nitrate assimilation is regulated in response to nitrate by a two-component system, encoded by the *nasTS* genes, which are located directly upstream of the *nasA* gene. NasS acts as a nitrate/nitrite sensor, whereas NasT has an ANTAR (AmiR and NasR transcription antitermination regulator) domain for acting as a transcriptional antiterminator [4,5]. In the absence of nitrate or nitrite, NasS and NasT form an inactive tetrameric complex (two units of each), leading to the premature termination of the *nasABGHC* gene transcription. In the presence of nitrate or nitrite, the NasTS complex becomes dissociated, and thereby NasT allows expression of *nas* genes [5]. Recently, the *P. dentrificans* PD1222 whole genome has been analyzed using the Quadparser programme, revealing the existence of a guanine-rich region located upstream of the *nasT* gene that forms a canonical G-quadruplex structure. Stabilization of this secondary structure in DNA has been suggested to act as a negative regulator for nitrate-dependent growth [6].

Nitrate assimilation is a widespread metabolic capacity in proteobacteria that usually is controlled at the transcriptional level by nitrate and nitrite induction and by ammonium repression [7,8]. In cyanobacteria, the catabolite activator protein (CAP) family transcription factor NtcA represses the nitrate reductase genes in the presence of ammonium, whereas it activates transcription of these genes at a high C/N ratio (nitrogen depletion), reflected by high 2-oxoglutarate levels [9–11]. In some cyanobacteria, the LysR family transcription regulator NtcB is required for the nitrate-/nitrite-dependent induction of the nitrate reductase gene [11]. In Gram-positive bacteria such as *Bacillus subtilis* and *Streptomyces coelicolor*, the TnrA and GlnR regulators respond to nitrogen starvation [12,13]. In *Klebsiella oxytoca*, expression of the nitrate assimilation genes is activated under low nitrogen conditions through the global nitrogen regulatory Ntr system, including NtrA, NtrB, and NtrC proteins, and by nitrate/nitrite induction through NasR, a transcription antitermination protein that also binds to nitrate [14,15]. The crystal structure of the *K. oxytoca* NasR protein has been solved; it is a dimer with a large N-terminal nitrate and nitrite-sensor (NIT) domain and a C-terminal ANTAR domain necessary for specific binding to leader mRNA. The NIT domain binds nitrate and nitrite between two conserved arginine residues located on adjacent helices [16]. In *Azotobacter vinelandii* and *Pseudomonas* *putida*, as well as in *P. denitrificans*, two different proteins NasT and NasS act as a transcriptional antiterminator and a nitrate sensor, respectively [17–20].

The NtrBC two-component system has been extensively characterized in enteric bacteria [21]. NtrB is a sensor kinase that autophosphorylates on a histidine residue under low nitrogen concentrations and transfers a phosphoryl group to the NtrC response regulator protein on a specific aspartate residue [22]. Phosphorylated NtrC acts as a transcriptional activator that oligomerizes on the DNA template with ATPase activity [23]. The NtrC members are usually dependent on the σ^54^ factor (NtrA), and they are involved in the transcription of genes related to nitrogen metabolism, such as the glutamine synthetase *glnA* gene. However, in *Rhodobacter capsulatus*, a regulatory two-component NtrBC system has been described in which the NtrC component is not σ^54^-dependent to activate transcription of *nifA1* and *nifA2* genes, which code for transcriptional activators that induce nitrogen fixation gene expression, and the *glnB* gene that is a negative regulator of the *R. capsulatus* NtrBC system under nitrogen excess [24].

The NtrYX system is also a two-component regulatory system with similarity to the sensor/kinase NtrB and the regulatory protein NtrC. This system has been investigated in diazotrophs with a proposed role in nitrogen assimilation. In *Azorhizobium caulinodans*, a mutant in the *ntrX* gene was found to be defective in using nitrate as the nitrogen source and showed also a reduced *nifA* expression under nitrogen fixation conditions with a disturbed symbiotic phenotype. In the *ntrC* mutant strain, expression of the *ntrYX* operon was derepressed in the presence of nitrate, suggesting an interaction between both NtrBC and NtrYX systems [25]. The NtrBC system in *Azospirillum brasilense* is involved in the regulation of nitrate assimilation, ammonium transport, and nitrogenase switch-off by ammonium. The NtrYX system may be involved in nitrate utilization through a possible substitution of the NtrBC system by the NtrYX sensor–regulator pair [26,27]. *Herbaspirillum seropedicae* is a diazotrophic β-proteobacterium with both NtrBC and NtrYX systems, displaying a role in the regulation of nitrate assimilation [28]. The photosynthetic bacterium *R. capsulatus* also has both NtrBC and NtrYX systems, with a function of the NtrBC system in urea assimilation and nitrogen fixation, but with an unclear physiological function of the NtrYX system [29]. The NtrYX system of *Brucella* spp. is involved in redox sensing and regulation of denitrification genes. Expression of *narGHIJK*, *nirKV*, *norBCDEFQ*, and *nosDFLRXYZ* genes is down-regulated in an *ntrY* mutant strain under aerobic and microaerobic conditions, with a marked down-regulation of the *nir*, *nor*, and *nos* genes under microaerobic conditions. The *Brucella* spp. NtrY protein contains one haem group for sensing the oxygen status in the cell and shows its maximal activity as an autohistidine kinase in the ferrous state under low oxygen tension [30,31].

Analysis of the whole genome sequence of *P. denitrificans* reveals the presence of genes encoding NtrBC and NtrYX proteins. In this work, we explore the global transcriptomic changes that underpin the transition from ammonium-dependent to nitrate-dependent aerobic growth, including the relationships within the different two-component regulatory systems NasTS, NtrBC, and NtrYX. An additional mechanism to the transcriptional regulation of nitrate assimilation, based on a nitrate-dependent translation control of the *P. denitrificans* PD1222 Nas proteins, is also proposed.

## Materials and methods

### Bacterial strains, media, and growth conditions

All strains used in the present study are listed in Supplementary Table S1. *P. denitrificans* PD1222 was grown at 30°C in Luria–Bertani (LB) medium [32] or defined mineral salts medium, as previously described [33], containing 50 mM succinate as the carbon source. Ammonium chloride, potassium nitrate, potassium nitrite, or l-glutamate were used as the nitrogen source (10 mM each), as stated in the text. Bacteria were cultured in 250 ml flasks containing 50 ml of medium that were rotated at 200 rpm. *Escherichia coli* strains were grown at 37°C in the LB medium. Cell growth was followed measuring the absorbance of the cultures at 600 nm (*A*_600_) or by protein determination [34]. Antibiotic supplements were used at the following concentrations (μg ml^−1^): ampicillin (Amp), 100; kanamycin (Km), 25; rifampicin (Rif), 100; spectinomycin (Sp), 25; streptomycin (Sm), 60; tetracycline (Tet), 10; and chloramphenicol (Cm), 50.

### Analytical methods

Upon reaching an exponential growth phase, a 50 ml culture volume was subject to cell fractionation. Cells were harvested, washed twice with 50 mM Tris–HCl (pH 8.0), and re-suspended in 10 mM Tris–HCl (pH 8.0), 500 mM sucrose, and 3 mM ethylenediaminetetraacetic acid (EDTA). Lysozyme (chicken egg white, EC 3.2.1.17) at 0.2 mg ml^−1^ final concentration and a few grains of DNase I (bovine pancreas, EC 3.1.21.1) were added. This mixture was incubated after shaking for 30 min at 30°C. The sphaeroplasts formed were harvested by centrifugation (13 000×***g***) for 15 min at 4°C and the periplasm was recovered. Sphaeroplasts were re-suspended in a volume of 10 ml in 100 mM Tris–HCl (pH 8.0) and disrupted by sonication, and cytoplasmic and membrane fractions were separated by ultracentrifugation (40 000×***g***). Assimilatory NADH-dependent nitrate reductase activity was assayed in cytoplasmic fractions in the presence of NADH as an electron donor by measuring the nitrite formed from nitrate [35]. β-Galactosidase activity was determined spectrophotometrically, as previously described [36]. Protein concentration was measured using a Bradford protein assay kit (Bio-Rad, U.K.) with bovine serum albumine as standard (Fraction V, Sigma, U.K.).

### Microarray analysis of *P. denitrificans*

*P. denitrificans* genomic DNA was isolated using a Genomic DNA Extraction Kit and 100/G columns (Qiagen) from 10 ml exponential phase cells, according to the specification of the manufacturer. For RNA extractions, 30 ml of early exponential phase cells (*A*_600_ ∼ 0.4) was added to 12 ml of ice-cold 95% ethanol/5% phenol (v/v) solution and incubated on ice for 30 min to prevent RNA degradation. Cells were then pelleted and stored at −80°C until RNA was extracted using the SV Total RNA isolation kit (Promega). Trace DNA contamination was removed by treatment with Turbo DNA-*free*™ DNase (Ambion), and this was confirmed by polymerase chain reaction (PCR) amplification of RNA samples using MyFi™ DNA polymerase (Bioline). Nucleic acids were quantified spectrophotometrically in a Nanodrop 2000 (Thermo Scientific), and the integrity of RNA samples was analyzed using an Experion™ Automated Electrophoresis platform (Bio-Rad) using RNA StdSens chips (Bio-Rad). All standard protocols were carried out according to the instructions of the manufacturers.

For labelling and hybridization of microarray slides, total RNA (10 µg) from three independent bacterial cultures was reverse-transcribed to cDNA using the AffinityScript multiple temperature reverse transcriptase (Agilent), and fluorescently labelled using random primers (Invitrogen) to incorporate Cy5-dCTP (deoxycytidine triphosphate containing Cy5 dyes; Amersham). *P. denitrificans* genomic DNA (10 µg) was labelled with Cy3-dCTP (deoxycytidine triphosphate containing Cy3 dyes; Amersham) using a Bioprime® DNA labelling system (Invitrogen), prior to mixing (1:5) with labelled cDNA, and hybridized to custom-designed 4 × 44 K oligonucleotide array slides (Agilent). Hybridization buffers [50 mM morpholine-4-ethanesulfonic acid (pH 6.5), 1 M NaCl, 20% (w/v) formamide, 20 mM EDTA, 1% (w/v) Triton X-100] mixed with Cy5- and Cy3-dCTP-labelled nucleic acids were loaded onto the GASKET slide prior to placing the microarray slide in contact with the hybridization mix, which were sealed in a tight chamber and incubated at 55°C in a rotary hybridization oven at 8 rpm, for ∼60 h in the dark. Following hybridizations, slides were removed and washed for 5 min in a microscope-slide chamber using a solution of 6× SSPE [0.2 M phosphate buffer, 2.98 M NaCl, and 20 mM EDTA (pH 7.4)] supplemented with 0.005% *N*-lauryl-sarcosine, followed by 5 min in a solution of 0.6× SSPE supplemented with 0.18% polyethylene glycol 200. Slides were then dried by centrifugation for 30 s.

**Figure 1.**
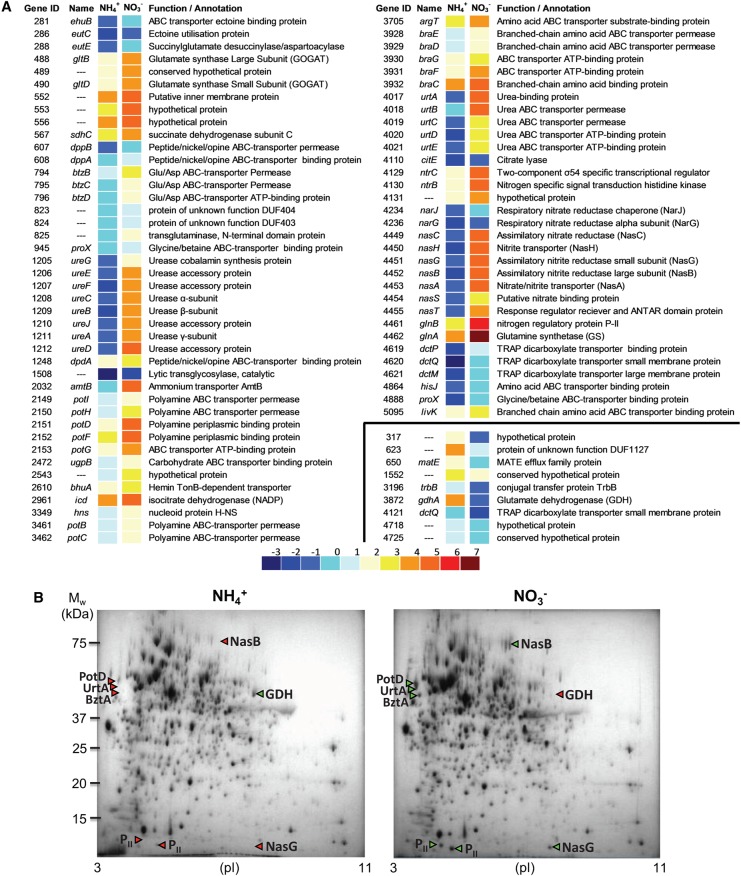
Transcriptomic and proteomic analysis of aerobically cells grown with nitrate or ammonium as the nitrogen source. (**A**) Heat-map from DNA microarray analyses of *P. denitrificans* PD1222 grown aerobically with either ammonium or nitrate as the sole nitrogen source. Colors indicate log_2_ of normalized expression values in either condition. Genes were selected based on at least 2-fold change in expression with *P*-values ≤0.05. A total of 74 genes were up-regulated >2-fold under nitrate assimilation conditions, whereas 9 genes were down-regulated >2-fold under these conditions. (**B**) 2D-PAGE analysis of *P.* *denitrificans* PD1222 soluble fractions from cells grown aerobically with ammonium or nitrate (10 mM each) as the sole nitrogen source. Spots present or absent from gels are highlighted by green or red triangles, respectively. The presented gels are representative of three independent replicants.

For analyses and interpretation, microarray slides were scanned using a scanner (GenePix 4000A, Axon Instruments) with excitation wavelengths of 532 and 635 nm. Fluorescent spots and background intensities were quantified using the GenePix Pro software (Axon Instruments) and filtered to omit those with a reference signal lower than 2 SD from the background intensity in further analyses. Signal intensities were corrected by subtracting the background and the red/green (Cy5/Cy3) ratios. All datasets were normalized using Batch Anti-Banana Algorithm in R (BABAR), which uses cyclic loess to normalize across the complete dataset [37], and analyzed using Gene Spring 7.3 (Agilent) to filter genes that were differentially expressed ≥2-fold with a significance of ≥95% across three independent cultures. These data are represented in Figure 1, where log_2_ of normalized expression values is shown as a heat-map. Microarrays were validated by quantitative polymerase chain reaction (qPCR) with oligonucleotide primers that annealed to internal regions of the *nas* genes (Supplementary Table S2), as described below. Primers were designed using the Primer^3^ Plus software [38], to amplify products between 100 and 150 bp, with a *T*_m_ of ∼60°C, and used at a final concentration of 0.4 µM. The relative fold-change values were calculated using amplification efficiencies, as described previously [39].

### Proteomic analysis

Two-dimensional polyacrylamide gel electrophoresis (2D-PAGE) was performed with sample preparations that were obtained from *P. denitrificans* cells grown to mid-log phase in minimal medium with different nitrogen sources, as previously described [2]. Subcellular fractionation was carried as described above, and isolated cytoplasmic fractions containing 250 μg of protein were used to rehydrate 11 cm strips (Immobiline DryStrips with the appropriate pH range; Amersham Biosciences) for 12 h. Isoelectric focusing was carried out in an IPGphor (Pharmacia) until 20 000 volt-h were reached. After isoelectric focusing, strips were equilibrated, as previously described [40], and applied to 12% (v/v) polyacrylamide gels. The second dimension (SDS-PAGE) was performed using the Hoefer SE600 system (Amersham Biosciences), and gels were stained using the Brilliant Blue G-colloidal concentrate (Sigma) and scanned with a Molecular Image FX (Bio-Rad). Triplicate 2D-PAGE separations were generated for each sample. Protein identification was carried out in the UCO-SCAI Proteomic Centre, University of Córdoba (Spain), a member of ProteoRed network. Protein spots of interest were excised automatically in a ProPic station (Genomic Solutions, U.K.), and samples were automatically digested with trypsin according to the standard protocols in a ProGest station (Genomic Solutions) and analyzed in a 4700 Proteomics Analyzer MALDI-TOF/TOF [matrix-assisted laser desorption/ionization (time of flight)] mass spectrometer (Applied Biosystems), in the *m*/*z* range of 800–4000, with an accelerating voltage of 20 kV, in reflectron mode and with delayed extraction set to ‘on’ and an elapsed time of 120 ns. Proteins were identified by peptide mass fingerprinting (PMF) and confirmed by MS/MS analysis of the three most abundant peptide ions. MASCOT searching engine (Matrix Science, U.K.) was used for protein identification over the non-redundant NCBI database of proteins. The confidence in the PMF matches (*P* < 0.05) was based on the molecular weight search (MOWSE) score (higher than 65) and CI >99.8%, and confirmed by the accurate overlapping of the matched peptides with the major peaks of the mass spectrum.

### Routine DNA manipulations and site-directed mutagenesis of *nasA* leader sequence

Genomic and plasmid DNA were routinely isolated and purified using the Wizard® Genomic DNA purification kit (Promega, U.S.A.) and the Qiagen plasmid kit (Qiagen, Germany), respectively. Custom oligonucleotide primers, listed in Supplementary Table S2, were supplied by Invitrogen (Paisley, U.K.), and the PCR was performed using the Expand High Fidelity PCR system (Roche, Switzerland) with 5% DMSO added as the standard.

In the mutational analysis carried out in *trans* of the *nasA* leader region, a *nasA* promoter transcriptional fusion was constructed using an intergenic *nasS*–*nasA* 392-bp fragment amplified by PCR with oligonucleotides FA1_A/RA1, which were located at the 3′-end of *nasS* and at the 5′-end of *nasA*. This fragment was cloned within the pSparkII vector and subcloned as *Sph*I–*Pst*I in the promoter probe vector pMP220, generating pMP220-*P*nasA (Supplementary Table S1). Different mutated versions of *P*nasA–*lacZ* were also constructed using oligonucleotides containing substitutions of three bases for two hairpins, HI and HII, identified in the *nasA* gene leader region (HI 137 bp and HII 91 bp upstream 5′-end *nasA* gene, respectively). Oligonucleotides FA1_B/RHI and FHII/RA1 were used for the mutagenesis of HI and HII, respectively, and the PCR products amplified with these oligonucleotides were cloned into the pSparkII vector. The pMP220-*P*nasA vector was digested with *Pst*I/*Asc*I and used to clone the mutated fragment of the HI previously liberated from pSparkII with *Pst*I/*Asc*I restriction enzymes, obtaining the pMP220-*P*nasA1 construct. The pMP220 plasmid is a non-integrative vector. For mutagenesis of HII, the corresponding mutated fragment was liberated from pSparkII with *Asc*I/*Sph*I and cloned into pMP220-*P*nasA previously digested with *Asc*I/*Sph*I, obtaining the pMP220-*P*nasA2 plasmid. All constructs were sequenced (UCO-SCAI, University of Córdoba) to confirm the native or the mutated sequences, and the final constructs were introduced into *P. denitrificans* PD1222 by triparental mating [2].

In the mutational analysis carried out in *cis* of the ANTAR region (HI), the DNA regions upstream and downstream this secondary structure were amplified by PCR with oligonucleotide pairs AIF1/AIR1a and AIF2/AIR2, respectively (Supplementary Table S2). These two fragments were cloned separately into the pSparkII cloning vector. The upstream fragment (524 bp) was subcloned as *Eco*RI/*Sal*I into pK18mobsacB, a kanamycin-resistant suicide vector in *P. denitrificans*. The downstream fragment (578 bp) was subcloned as *Sal*I/*Hin*dIII into the previous construction. The final construction contained a 35 bp sequence corresponding to the multicloning site of the pSparkII vector, replacing the native hairpin I sequence (37 bp). This plasmid was used for triparental mating with the receptor strain *P. denitrificans* and the helper strain *E. coli* containing the pRK2013 plasmid. First transconjugant selection was carried out in the LB medium with spectinomycin and kanamycin. After that, a sucrose selection for double-crossover events was performed. Finally, mutation was confirmed by PCR and enzyme restriction analyses. The wild-type *nasA* leader region with HI was amplified with oligonucleotides FA1_A/RA1. This wild-type fragment was not digested by *Sal*I, whereas the fragment amplified from the mutant was digested by this restriction enzyme, because it contains the *Sal*I recognition sequence of the pSparkI multicloning site.

### Site-directed mutagenesis of the NtrC-binding sequence

The native *nasT* promoter, as well as a mutated version affected in the putative NtrC-binding site located upstream of the *nasT* gene, was used to generate transcriptional fusions by PCR-driven overlap extension. Initial PCRs were carried out with oligonucleotides PTF/PTNtrR and PTR/PTNtrF for the native construct, and PTF/PTNtrR_Mut and PTR/PTNtrF_Mut for the mutated version. Internal primers (PTNtr) were used to generate overlapping and complementary 3′-ends on the intermediate segments (Supplementary Table S2). In the case of the mutated *nasT* promoter, the PTNtr primers were also used to introduce the mutated bases. Overlapping strands hybridize at this region in a subsequent PCR and were extended to generate the full-length product (619-bp) amplified by flanking primers that include restriction enzyme sites for cloning as *Pst*I/*Sph*I into the promoter probe vector pMP220. The final constructs for the analysis of the native *nasT* promoter and the NtrC-binding site mutated version of the *nasT* promoter were called pMP220-*P*nasT and pMP220-*P*nasT1, respectively. All plasmids were checked by sequencing (UCO-SCAI, University of Córdoba), and the final constructs were introduced into *P. denitrificans* PD1222 by triparental mating [2].

### Reverse transcription polymerase chain reaction and quantitative polymerase chain reaction

*P. denitrificans* wild-type and mutant strains were cultured in minimal medium with succinate as the carbon source and different nitrogen sources. Cells were harvested (*A*_600_ ∼ 0.4) and washed in TEG buffer with 25 mM Tris–HCl (pH 8.0), 1% glucose, and 10 mM EDTA. RNA isolations were performed following the Qiagen RNA extraction kit (RNeasy midi kit). DNase incubation was carried out in the column with the RNase-free DNase set (Qiagen) and an additional post-column treatment was required with DNase I (Ambion). The concentration and purity of the RNA samples were measured in an ND1000 spectrophotometer (Nanodrop Technologies). Synthesis of total cDNA was achieved in 20 µl final volume, containing 500 ng of RNA, 0.7 mM dNTPs, 200 U SuperScript II Reverse Transcriptase (Invitrogen), and 3.75 μM random hexamers (Applied Biosystems). Samples were initially heated at 65°C for 5 min and then incubated at 42°C for 50 min, followed by incubation at 70°C for 15 min. To carry out PCRs, 2 μl of each cDNA was initially heated at 98°C for 2 min, followed by 30 cycles of amplification: 98°C, 30 s; 60°C, 30 s, and 69°C, 1 min. Polymerase extension reactions were completed by an additional incubation at 69°C for 10 min. For real-time assays, the cDNA was purified using a Favorprep Gel/PCR purification kit (Favorgen) and the concentration was measured using an ND1000 spectrophotometer. The iQ5 Multicolor Real-Time PCR Detection System (Bio-Rad) was used in a 25 μl reaction (final volume), containing 2 μl of diluted cDNA (12.5, 2.5, and 0.5 ng), 0.2 μM of each primer (Supplementary Table S2), and 12.5 μl of iQ SYBR Green Supermix (Bio-Rad). Target cDNAs and reference samples were amplified three times in separate PCRs. Samples were initially denatured by heating at 95°C for 3 min, followed by 40 cycles of amplification (95°C, 30 s; test annealing temperature, 60°C, 30 s; elongation and signal acquisition, 72°C, 30 s). For relative quantification of the fluorescence values, a calibration curve was made using dilution series from 100 to 0.001 ng of *P. denitrificans* genomic DNA sample. Represented data were normalized using the *rpoB* and the *dnaN* genes as housekeeping (Supplementary Table S2). Primer design and relative fold-change value calculation were carried out, as previously described [38,39].

### Generation of *ntrB* and *ntrY* mutant strains of *P. denitrificans*

Mutant strains of *P. denitrificans* were generated by significant allelic replacement with specific antibiotic resistance markers. The *ntrB* mutant (*ntrB*Δ::Sm) was generated by PCR amplification of the 3′-end (682 bp) and 5′-end (533 bp) of the *ntrB* gene with the respective set of primers ntrB1/ntrB2 and ntrB3/ntrB4 (Supplementary Table S2). The PCR products were cloned within the pGEM-T Easy vector and then assembled within the pUC18 vector (Supplementary Table S1). A unique *Bam*HI restriction site was generated at the interface of the 3′-end and the 5′-end of *ntrB* gene to generate a 685 bp deletion and to insert a streptomycin resistance cassette from the pSRA2 vector (Supplementary Table S1). A single *Eco*RI fragment (*ntrB*Δ::Sm) was transferred to pSUP202 to produce the final mobilizable vector.

For the *ntrY* mutant strain (*ntrY*Δ::Km), fragments containing the 3′-end (510 bp) and 5′-end (717 bp) of *ntrY* were produced by PCR using the respective primer pairs ntrY1/ntrY2 and ntrY3/ntrY4 (Supplementary Table S2), and cloned within the pGEM-T vector (Supplementary Table S1). The upstream region was screened by restriction digestion and selected using an *Eco*RI recognition site, and the downstream region of *ntrY* was introduced as an *Aat*II–*Bam*HI fragment, yielding a unique *Bam*HI restriction site between both regions, generating a 1035 bp deletion. A kanamycin resistance cassette obtained from the pSRA2 vector (Supplementary Table S1) was inserted as a *Bam*HI fragment between these regions of *ntrY*. Finally, the *Aat*II–*Eco*RI fragment (*ntrY*Δ::Km) was cloned into the mobilizable vector pSUP202. This construct was transferred to either the wild-type strain or the *ntrB* mutant to generate the single *ntrY* or the double NtrB/NtrY mutant, respectively. Plasmids were analyzed by DNA-sequencing (UCO-SCAI, University of Córdoba), and mutants were checked by PCR using primer pairs that amplify for a product containing the deletion–insertion mutation. Mutants showed bands higher than those presented in the wild type because the inserted resistance cassettes were larger than the deleted fragments, confirming allelic exchange by a double-crossover mechanism.

## Results

### Transcriptomic differences between bacteria cultured aerobically under nitrate- versus ammonium-dependent anabolism

To investigate the underlying biochemical adaptation to nitrate assimilation compared with ammonium-dependent anabolism, transcriptomic analysis was undertaken through microarray analysis of RNA isolated from *P. denitrificans* cells grown in batch culture with 20 mM succinate and either 10 mM nitrate or 10 mM ammonium as the sole N-source. Of the 5077 protein-coding genes that are present in the genome of *P. denitrificans* PD1222, only 83 genes (1.64% of the whole genome) displayed at least 2-fold expression differences at a 95% significance level between the two growth conditions tested. Among these, 74 genes were induced during nitrate-dependent growth, while 9 genes were down-regulated (Figure 1A).

The genes induced when nitrate was the only nitrogen source belonged to 30 gene clusters, including among others, the *gltB* and *gltD* genes encoding the small and large subunits of the glutamate synthase (GOGAT), the *amtB* gene that codes for a high-affinity ammonium transporter, the *ntrB* and *ntrC* genes that code for the general nitrogen regulatory Ntr system, the *glnB* gene that codes for the nitrogen regulatory protein PII, the *glnA* gene encoding the glutamine synthetase, the *bztA-D* gene cluster involved in glutamate/aspartate transport, the *ureA-GJ* gene cluster for urea metabolism, the *urtA-E* gene cluster for urea transport, the *potDF-I* gene cluster involved in polyamine transport, the *braC-G* gene cluster involved in branched-chain amino acids transport, the *dctPQM* genes encoding a tripartite ATP-independent periplasmic (TRAP) dicarboxylate transporter system, the respiratory nitrate reductase *narGJ* genes, and the *nasTSABGHC* gene cluster for nitrate/nitrite assimilation (Figure 1A). The genes down-regulated during growth with nitrate included the *gdhA* gene that encodes the glutamate dehydrogenase (Figure 1A). Expression of a selection of these genes was also quantified by qPCR and corroborated well with the microarray data (Table 1). The genes for which expression was highly increased on nitrate included *nasA*, *nasH*, *narG*, *narJ*, *nasT*, *nasS*, *urtC*, *amtB*, *ureD*, *glnA*, *glnB*, *gltD*, and *ntrC.* The *nasT* gene was more strongly induced by nitrate than the *nasS* gene (Table 1).

**Table 1:** Analysis by RT-qPCR of nitrate-induced genes detected in the microarrays of *P. denitrificans* Data correspond to the mean ± standard deviation of the nitrate/ammonium gene expression ratio.

Gene		NO_3_^−^/NH_4_^+^
Pden_0490	*gltD*	2.8 ± 0.5
Pden_0794	*bztB*	1.9 ± 0.4
Pden_1212	*ureD*	5.1 ± 1.8
Pden_2032	*amtB*	5.3 ± 1.7
Pden_2151	*potD*	2.6 ± 0.2
Pden_4019	*urtC*	6.2 ± 0.4
Pden_4129	*ntrC*	4.6 ± 0.5
Pden_4130	*ntrB*	1.6 ± 0.2
Pden_4234	*narJ*	24.3 ± 11.1
Pden_4236	*narG*	100.5 ± 23.3
Pden_4450	*nasH*	185.7 ± 9.8
Pden_4453	*nasA*	385.0 ± 11.9
Pden_4454	*nasS*	7.7 ± 1.5
Pden_4455	*nasT*	23.3 ± 6.3
Pden_4461	*glnB*	2.6 ± 0.6
Pden_4462	*glnA*	3.4 ± 0.7

Qualitative proteomic analysis by 2D-PAGE of soluble fractions of *P. denitrificans* cells grown with nitrate or ammonium as the sole N-source confirmed that a significant number of the key changes in the transcriptome could be also identifed in the proteome (Figure 1B). The up-regulated proteins for which higher levels of synthesis in the presence of nitrate could be confirmed included the nitrogen regulatory protein GlnB (PII), the periplasmic component of the urea ABC-type transporter (UrtA), the polyamine transporter component (PotD), the glutamate/aspartate transporter component (BztA), and two proteins encoded by the *nas* gene cluster of *P. denitrificans* (NasG and NasB). A decreased synthesis of the glutamate dehydrogenase (GdhA) was observed in the nitrate-grown cells compared with the ammonium-grown cells (Figure 1B).

### Analysis of the expression of the *nasTS* and *nasABGHC* transcripts

**Figure 2.**
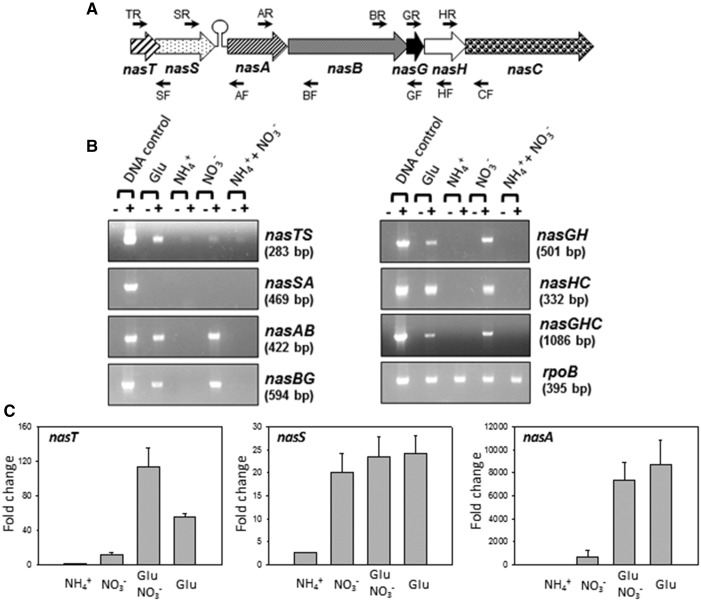
Analysis by RT-PCR and qPCR of the *P. denitrificans* nitrate assimilation *nas* genes. (**A**) The *nas* gene cluster of *P. denitrificans* and the location of the oligonucleotides used in the present study are shown (see Supplementary Table S2). (**B**) RT-PCRs with RNA from the wild-type strain of *P. denitrificans* grown in the presence of different N-sources. To isolate RNA, cells were cultured in minimal medium with different N-sources, as described in the Materials and Methods section and harvested at an *A*_600_ of ∼0.4. The *rpoB* gene was used as housekeeping. −/+, without/with reverse transcriptase. (**C**) RT-qPCR analysis of the *nasT*, *nasS*, and *nasA* genes from cells grown with different nitrogen sources. Error bars represent standard deviation calculated from the results of three independent experiments.

The transcriptome differences between nitrate- and ammonium-anabolizing cells suggest a general adaptation to the metabolism of secondary (non-ammonium) nitrogen sources, nitrogen scavenging, and the need to search for organic carbon to provide the reductant for nitrate assimilation. Alongside, this is a more specific adaptation to nitrate anabolism through expression of the *nasABGHC* genes. Therefore, the control of the *nas* gene expression was investigated. The regulatory pattern for *P. denitrificans nas* gene expression was analyzed at the transcriptional level by RT-PCR analysis with RNA from cells grown with nitrate, glutamate, ammonium, or ammonium plus nitrate as the N-source. Primer pairs were designed to explore transcription across the 3′- and the 5′-ends of different *nas* gene boundaries (Figure 2A and Supplementary Table S2). The PCR products were detected across the *nasTS*, *nasAB*, *nasBG*, *nasGH*, *nasHC*, and *nasGHC* boundaries (Figure 2B). However, PCR products were undetectable when the primers located at the 3′-end of the *nasS* gene and the 5′-end of the *nasA* gene were used. These results suggest that the *nasTSABGHC* gene cluster comprises two different transcriptional units, one corresponding to the regulatory *nasTS* genes and the other constituted by the structural *nasABGHC* genes.

The RT-PCR analysis indicated that the *nasA-C* transcript is found in cells grown with either nitrate or glutamate as the sole N-source, but not when ammonium was present (Figure 2). However, a high glutamine concentration (5 mM) or a 2-oxoglutarate/glutamine 5 mM/5 mM ratio did not repress expression of the *nas* genes. The *nasA* gene expression, determined by RT-qPCR, was similar in cells grown with glutamate or glutamate plus nitrate (Figure 2C). The *nasTS* transcript was detected in cells grown with all nitrogen sources tested, although only at very low levels in the presence of ammonium (Figure 2C).

**Figure 3.**
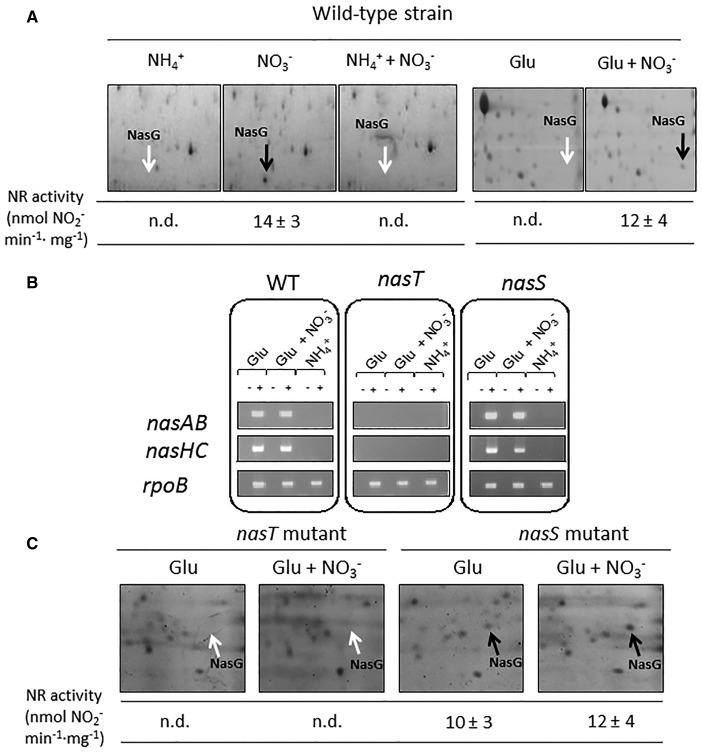
Characterization of the wild-type strain and the *nasT* and *nasS* mutants of *P. denitrificans* with different nitrogen sources. (**A**) NADH-dependent nitrate reductase activity and NasG polypeptide detection by 2D-PAGE analysis in the wild-type strain of *P. denitrificans*. The 2D-PAGE analyses were carried out in cytoplasmic fraction from cells grown with different nitrogen sources obtained by subcellular fractionations, as indicated in Materials and Methods. The different nitrogen sources were nitrate, ammonium, ammonium plus nitrate, glutamate, or glutamate plus nitrate (10 mM each). Isoelectric focusing was performed from IPG strips (range 4–7) and second dimension was carried out onto 12% polyacrylamide gels. The nitrate reductase activity (NR) was assayed in cytoplasmic fractions and expressed as nmol NO_2_^−^ formed min^−1^ mg^−1^ (n.d., not detected). (**B**) RT-PCR analysis of structural *nas* genes in the wild-type and *nasT* and *nasS* mutant strains of *P. denitrificans*. To isolate RNA, wild-type and *nasT* and *nasS* strains were cultured in minimal medium with different N-sources, as described in the Materials and Methods section and harvested at an *A*_600_ of ∼0.4. The *rpoB* gene was used as housekeeping. −/+, without/with reverse transcriptase. (**C**) NasG polypeptide detection by 2D-PAGE analysis and NADH-nitrate reductase activity in cytoplasmic fractions from *nasT* and *nasS* mutant strains of *P. denitrificans.* The 2D-PAGE analysis and the NR activity assays were performed as indicated in (**A**) for the wild-type strain.

A proteomic approach was applied to confirm the presence of polypeptides encoded by the *nas* genes in cytoplasmic fractions of *P. denitrificans* cells grown with different nitrogen sources. The Rieske-type protein NasG required for both nitrate and nitrite reductase activities (Figure 3A) and the catalytic subunit of the nitrite reductase NasB (not shown) were detected only when nitrate was present in the media and ammonium was absent. NADH-dependent nitrate reductase activity was determined in the cytoplasmic fraction from cells grown with different nitrogen sources. In accordance with the proteomic analysis, the nitrate reductase activity was detected only in the presence of nitrate and the absence of ammonium (Figure 3A). It is notable that although the *nasABGHC* transcript was detected in cells grown with glutamate in the absence of nitrate (Figure 2B), the NasG and NasB proteins and the nitrate reductase activity were not detectable under these conditions (Figure 3A).

### Analysis of *nas* gene expression in the *nasT* and *nasS* mutants

It has been previously described that a *nasT* mutant of *P. denitrificans* is unable to grow with nitrate as the sole N-source, whereas a *nasS* mutant of *P. denitrificans* grows with nitrate as the sole N-source with similar rates and yields to that shown by the wild-type strain [5]. To analyze the regulatory effect of nitrate in the *nasS* and *nasT* mutants, a comparison of *nas* gene expression between cells grown with glutamate versus cells grown with glutamate plus nitrate was performed. RT-qPCR analysis showed that the *nasT* mutant lacks the *nasABGHC* transcript (Figure 3B). Accordingly, 2D-PAGE revealed that this mutant strain is also devoid of the NasG polypeptide (Figure 3C). It is noticeable that the respiratory nitrate reductase *narG* and *narJ* genes were up-regulated in the *nasT* mutant strain, whereas genes coding for glutamine synthetase (*glnA*) and PII regulatory protein (*glnB*) were down-regulated (Table 2), suggesting that NasT might also serve as part of a more complex global regulatory system.

**Table 2:** Analysis by RT-qPCR of nitrate-induced genes in the wild-type, *nasT*, and *nasS* mutant strains of *P. denitrificans* Data correspond to the mean ± standard deviation of the glutamate plus nitrate/glutamate gene expression ratio.

Gene		Wild-type Glu + NO_3_^−^/Glu	*nasT* mutant Glu + NO_3_^−^/Glu	*nasS* mutant Glu + NO_3_^−^/Glu
Pden_0490	*gltD*	0.6 ± 0.1	0.7 ± 0.1	2.6 ± 0.2
Pden_0794	*bztB*	2.2 ± 1.6	0.7 ± 0.4	–
Pden_1212	*ureD*	1.1 ± 0.4	1.0 ± 0.1	1.8 ± 0.1
Pden_2032	*amtB*	0.4 ± 0.0	0.6 ± 0.1	0.3 ± 0.1
Pden_2151	*potD*	0.5 ± 0.1	0.6 ± 0.1	–
Pden_4019	*urtC*	0.5 ± 0. 1	0.5 ± 0.1	–
Pden_4129	*ntrC*	0.4 ± 0.1	0.5 ± 0.1	0.8 ± 0.2
Pden_4130	*ntrB*	0.7 ± 0.1	0.6 ± 0.2	0.6 ± 0.1
Pden_4234	*narJ*	7.7 ± 1.2	57.0 ± 14.4	–
Pden_4236	*narG*	2.5 ± 0.2	67.5 ± 12.2	173.8 ± 12.3
Pden_4450	*nasH*	0.6 ± 0.2	0.9 ± 0.1	0.2 ± 0.1
Pden_4453	*nasA*	0.9 ± 0.3	0.3 ± 0.1	0.2 ± 0.1
Pden_4454	*nasS*	1.0 ± 0.3	1.4 ± 0.1	0.0 ± 0.0
Pden_4455	*nasT*	2.0 ± 0.4	0.0 ± 0.0	1.8 ± 0.1
Pden_4461	*glnB*	2.0 ± 0.2	0.4 ± 0.1	1.0 ± 0.1
Pden_4462	*glnA*	1.7 ± 0.1	0.4 ± 0.1	1.2 ± 0.1
Pden_3872	*gdhA*	2.0 ± 0.3	1.1 ± 0.2	11.4 ± 1.0

In the *P. denitrificans nasS* mutant, the *nasABGHC* transcript was detected in all nitrogen sources, except when ammonium was used as the N-source, similar to the wild-type strain (Figure 3B). The NasG polypeptide and the nitrate reductase activity were detected in the *nasS* mutant strain grown with glutamate both in the presence and absence of nitrate (Figure 3C), whereas in the wild-type strain the NasG polypeptide and the nitrate reductase activity were detected only in the presence of nitrate (Figure 3A). In addition, RT-qPCR analysis revealed that the *nasS* mutant strain showed significantly increased levels of the *narG* and *gdhA* genes in response to nitrate (Table 2).

### Identification of putative *cis*-regulatory RNA secondary structures in the *nasA* leader region

**Figure 4.**
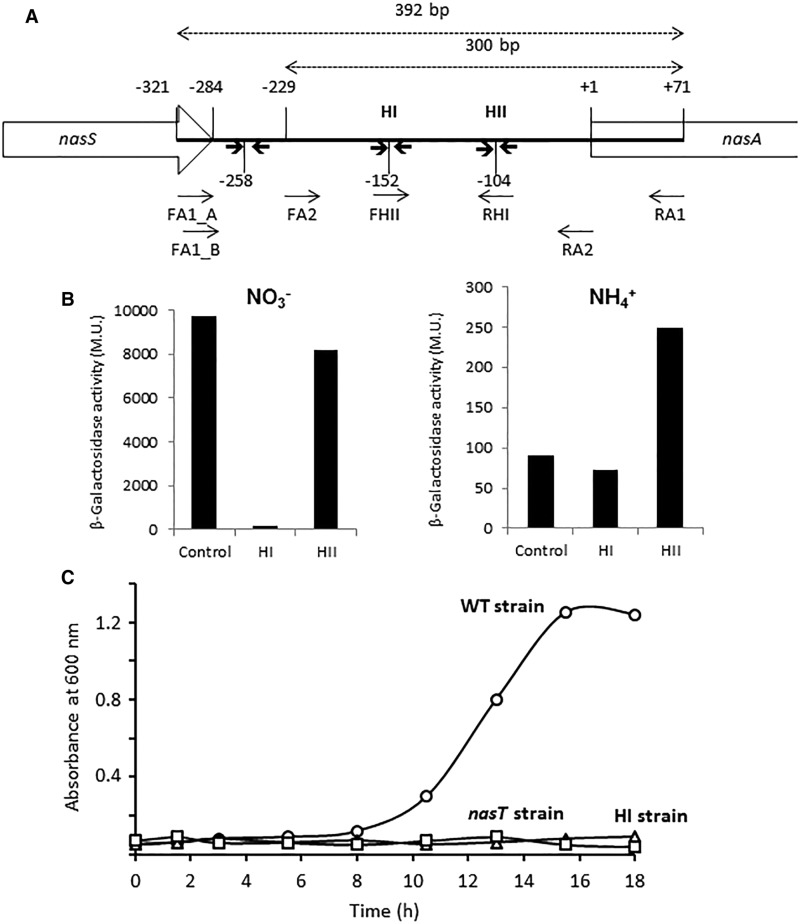
Analysis of the *nasA* leader region of *P. denitrificans*. (**A**) Intergenic *nasS*–*nasA* region from *P. denitrificans*. (**B**) Expression of the transcriptional PnasA–*lacZ* fusions and effect of mutations within the leader sequence on *lacZ* expression in cells grown with ammonium or nitrate (10 mM each). The β-galactosidase activity was measured when the *A*_600_ was ∼1.0 and it was represented as Miller units (M.U.). Control represents the wild-type (native) construction from −321 to +71 nucleotides of the *nasS–nasA* intergenic region. HI and HII represent the PnasA–*lacZ* constructions with 3 bp mutations in each hairpin. (**C**) Growth curves with nitrate as the sole nitrogen source of the *P. denitrificans* wild-type strain and the mutants in the *nasT* gene and the *cis*-acting NasT-binding region (hairpin I, HI).

Between the regulatory *nasTS* genes and the structural *nasABGHC* genes lies a ∼200 bp non-coding region with putative hairpin structures that could lead to transcription termination (Figure 4A). Secondary structures in the RNA leader sequence of *nasA* are thought to terminate transcription [14,15], but binding of NasT could allow transcription antitermination. Putative hairpin structures that could be a target for the transcriptional antiterminator NasT in the *nasA* leader region (PnasA) were identified using the RNAfold web server (http://rna.tbi.univie.ac.at/cgi-bin/RNAfold.cgi). To explore the importance of this region, several PnasA-*lacZ* transcriptional fusions were constructed and expressed in *P. denitrificans* cells grown with either nitrate or ammonium as the sole N-source. In cells grown under nitrate anabolizing conditions, the mutation of the HI hairpin resulted in a severe deficiency in the transcriptional activity. However, transcriptional activity was relatively unaffected in the HII mutation (Figure 4B). Cells grown with ammonium had the transcriptional activity 100-fold lower than nitrate-assimilating cells, but there was a 2-fold increase in cells carrying the HII mutation. These results suggest that the HI hairpin is important for NasT binding, and that HII structure may contribute to transcription attenuation. To corroborate the importance of HI, a specific chromosomal mutation in *cis* of the putative NasT-binding region was introduced. This chromosomal mutant lost the parental capacity to grow with nitrate as the sole nitrogen source, as the mutant in the *nasT* gene (Figure 4C). Controls established that the chromosomal HI mutant strain showed a similar growth to the parental strain with ammonium as the nitrogen source.

### The contribution of *ntrBCYX* to the regulation of nitrate assimilation

In addition to *nasTS* genes, the *ntrBC* genes were also up-regulated during nitrate-dependent anabolism compared with cells grown with ammonium (Figure 1A and Table 1). This suggests an integrated regulation of nitrate metabolism between these two systems. The *ntrBC* genes are clustered together with the *ntrYX* genes. The *ntrB* gene encodes a signal transduction protein (386 residues) with histidine kinase activity involved in nitrogen metabolism, and the *ntrC* gene codes for a Fis-like σ^54^-dependent regulator receiver protein (486 residues). Similarly, the *ntrY* gene codes for a multi-sensor signal transduction regulator (709 residues) with histidine kinase activity, and the *ntrX* gene encodes a σ^54^-dependent regulatory protein (463 residues). A non-coding region of 217 bp lies between *ntrC* and *ntrY* genes, whereas *ntrY* and *ntrX* genes overlap by 3 bp. NtrB and NtrC homologues have been found in many different microorganisms (Supplementary Figure S1). Although the NtrYX system is not widely distributed among bacteria, the *P. denitrificans* NtrY protein showed the highest identity (41%) with *A. caulinodans* NtrY, whereas the *P. denitrificans* NtrX protein showed the highest identity (56%) with its homologues in *A. caulinodans* and *Azospirillum brasilense* (Supplementary Figure S2).

**Figure 5.**
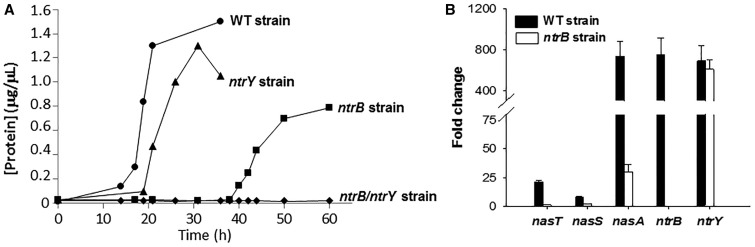
Characterization of the *ntrB*, *ntrY*, and double *ntrB*/*ntrY* mutant strains of *P. denitrificans* in media with nitrate. (**A**) Growth curves of *P. denitrificans* wild-type strain (●), and *ntrB* (▪), *ntrY* (▴), and double *ntrB*/*ntrY* (◆) mutants with 10 mM nitrate as the nitrogen source. (**B**) RT-qPCR analysis of the regulatory genes *nasT*, *nasS*, *ntrB*, and *ntrY* and the structural *nasA* gene in the wild-type strains and the *ntrB* mutant strains of *P. denitrificans* grown with 10 mM nitrate as the N-source. The oligonucleotides used in the qPCR analysis for the *ntrB* and *ntrY* genes hybridized upstream the mutation site. Represented data were normalized using the *rpoB* and *dnaN* genes as housekeeping (Supplementary Table S2). Error bars represent standard deviation calculated from the results of three independent experiments.

Isolation and characterization of single *ntrB* and *ntrY* mutants and double *ntrB*/*ntrY* mutant of *P. denitrificans* were carried out. All mutant strains displayed similar growth rates and yields to that shown by the wild-type strain when ammonium was used as the nitrogen source. However, the *ntrY* mutant was only slightly affected on its growth on nitrate, whereas the *ntrB* mutant showed a longer lag-phase than the wild-type mutant, and the double *ntrB*/*ntrY* mutant was unable to grow with nitrate (Figure 5A).

Expression of the *nasT*, *nasS*, *ntrB*, and *ntrY* regulatory genes and the structural *nasA* gene were analyzed by RT-qPCR in the *ntrB* and *ntrY* mutants and the wild-type strain of *P. denitrificans*. In the *ntrB* mutant, *nasTS* and *nasA* were down-regulated (Figure 5B), whereas in the *ntrY* mutant all these genes were expressed similar to the wild-type strain (not shown). The assimilatory NADH-dependent nitrate reductase activity was determined in cytoplasmic fractions from wild-type, *ntrB* and *ntrY* mutants grown with nitrate, when all strains reached similar biomass (protein concentration ∼150 mg ml^−1^). The *ntrY* mutant showed similar levels of activity to that presented by the wild-type mutant, ∼4 nmol NO_2_^−^ formed min^−1^ mg^−1^, whereas the *ntrB* mutant presented only an activity of ∼1 nmol NO_2_^−^ formed min^−1^ mg^−1^.

Transcriptional fusions to the β-galactosidase-encoding gene *lacZ* were carried out in the *nasT* leader sequence (PnasT) and expressed in the wild-type, *ntrB*, and *ntrY* mutant strains of *P. denitrificans*. The β-galactosidase activities in the *ntrY* mutant were very similar to those found in the wild-type strain (Table 3). However, β-galactosidase activities in the *P. denitrificans ntrB* mutant were much lower than those described for the wild-type strain (Table 3).

**Table 3:** Analysis of the *nasT* promoter by *lacZ* (β-galactosidase activity) gene fusion in *P. denitrificans* wild-type and *ntrB* and *ntrY* mutant strains

Strain/construct	N-source			
	NH_4_^+^	NO_3_^−^	Glu	Glu + NO_3_^−^
WT/PnasT (native)	71 ± 5	14 500 ± 143	11 000 ± 120	15 000 ± 152
WT/PnasT (mutated)*	50 ± 3	220 ± 14	53 ± 5	600 ± 25
*ntrY*/PnasT (native)	n.d.	n.d.	11 500 ± 115	15 200 ± 135
*ntrB*/PnasT (native)	n.d.	n.d.	10 ± 1	120 ± 4

* The base substitutions are specified in Supplementary Table S2. Abbreviations: n.d., not determined.

These data strongly suggest a role for NtrBC in the regulation of nitrate assimilation at the level of expression of *nasT*, and so, the possibility of a *cis*-acting binding site for NtrC was explored. The RegPrecise program for collection of manually curated inferences of regions in prokaryotic genomes identified a very well-conserved putative NtrC-binding site [5′-(T/C)GC(C/A)NNNNTTNNT(G/A)GCA-3′] in the leader regions of many of the different genes in *P. denitrificans*, including the *glnBA*, *amtB*, and *nasTS* genes. These genes were up-regulated during nitrate assimilation, as revealed by the transcriptomic analyses (Figure 1A and Table 1). Several base pairs were mutated in the putative NtrC-binding site located in the *nasT* leader sequence, resulting in the loss of the up-regulation observed under nitrate- or glutamate-dependent growth conditions (Table 3). These results highlight the importance of this sequence motif in the regulation of nitrate assimilation.

## Discussion

The soil denitrifier *P. denitrificans* can use nitrate as the sole N-source by an assimilatory nitrate reduction system. The process of nitrate assimilation in this bacterium has been comprehensively investigated by comparative transcriptomic and proteomic analyses in cells grown under nitrate- or ammonium-assimilating conditions (Figures 1 and 3 and Supplementary Figure S3). Comparing nitrate- with ammonium-grown cells, a key metabolic change was the decrease in the expression of glutamate dehydrogenase, the enzyme related with the low-affinity ammonium assimilation pathway. This was accompanied by the induction of components for organic nitrogen scavenging, urea uptake and utilization, high-affinity ammonium uptake and assimilation, regulatory and structural components of inorganic nitrate and nitrite assimilation, and scavenging of dicarboxylate organic acids to provide the additional reducing equivalents required for nitrate assimilation (eight electrons per nitrate ion converted into ammonia).

In *P. denitrificans*, the regulatory *nasTS* genes are located at the 5′-end of the *P. denitrificans nas* gene cluster for nitrate assimilation (Figure 2A). RT-PCR and qPCR analyses of the *P. denitrificans nasTS* and *nasABGHC* genes demonstrate that these genes are transcribed as separated transcriptional units. The existence of two independent transcriptional units in the *P. denitrificans nas* region is also supported by the presence of a non-coding region between the regulatory *nasTS* genes and the structural *nasABGHC* genes, with two putative hairpins (HI and HII) that could lead to secondary structures in this mRNA region (Figure 4). These two transcriptional units show different regulations, since expression of the regulatory *nasT* and *nasS* genes was observed in all N-sources tested, including ammonium, although in general at very low levels, whereas the *nasABGHC* transcript was detected either with nitrate or with glutamate, but it was undetectable with ammonium independently of the presence or absence of nitrate (Figure 2). However, although the *nasABGHC* transcript was present in wild-type cells grown with glutamate as the sole N-source, the Rieske-type NasG protein and the large subunit of the assimilatory nitrite reductase NasB, required for both nitrate and nitrite reductase activities, were not detected by 2D-PAGE, and these proteins were observed only in the presence of nitrate. Accordingly, the assimilatory NADH-dependent nitrate reductase activity was not detected when glutamate was used as the sole nitrogen source (Figure 3A). These results suggest that nitrate is not an obligate inducer of the *nasABGHC* gene expression, and that it may also play a positive role at the translational level.

Mutational analysis of the *nasT* gene of *P. denitrificans* reveals that NasT could act as a transcription antiterminator, because the *nasT* mutant strain is unable to grow with nitrate as the sole N-source and lacks both *nasABGHC* transcript and NADH-nitrate reductase activity (Figure 3B,C). Also, the strain harbouring the chromosomal mutation in a putative *cis*-acting NasT-binding site (HI) was unable to grow with nitrate (Figure 4C). Therefore, in the present work, we have identified the RNA region where NasT is likely to bind as a transcriptional antiterminator. On the contrary, the *nasS* mutant strain of *P. denitrificans* is capable of using nitrate as the sole N-source, similar to the wild-type strain, but shows a deregulated nitrate reductase activity that can be detected independently of the presence or absence of nitrate (Figure 3B,C). In addition, assimilatory nitrate reductase activity in the *P. denitrificans nasS* mutant is abolished in the presence of ammonium, as previously described in the wild-type strain [5]. Expression of the *nasT* gene is higher than *nasS* gene expression (Tables 1 and 2), probably because strong secondary structures present at the 5′-end of the *nasS* gene might provoke RNA polymerase to be occasionally released without completing the whole transcript.

*P. denitrificans* NasS and NasT proteins expressed in *E. coli* have been purified as a tetrameric complex in the absence of nitrate or nitrite, but in addition to the NasT–NasS complex, free NasT protein can be also detected [5]. Therefore, in the absence of nitrate, NasS and NasT form a protein–protein complex, probably with two units of each protein, which limits binding of NasT to the *nas* mRNA to act as a transcription antiterminator. However, in the presence of nitrate, NasS clamps the oxyanion, changing conformation and dissociates from NasT, thus increasing the size of the free NasT pool available to serve for *nasABGHC* transcription antitermination [5]. However, the elevated *nasT* gene expression over the *nasS* gene could lead to an excess of NasT protein over the NasS protein. This unbalance implies that there is always enough free NasT to allow transcription of *nasABGHC* genes in the absence of both nitrate and ammonium (i.e. glutamate as the sole N-source), suggesting additional global regulatory roles. In the *P. denitrificans nasT* mutant, the structural *nasABGHC* transcript was absent from all N-sources tested (Figure 3B). This is consistent with a role for NasT as a transcription antiterminator and with the phenotype of the *P. denitrificans nasT* mutant, which is unable to grow with nitrate or nitrite as the sole N-source. In addition, up-regulation of *narG* and *narJ* genes and down-regulation of *glnAB* genes in the *nasT* mutant also indicated that NasT may be involved in other regulatory processes. Recently, it has been suggested in the soybean endosymbiont *Bradyrhizobium japonicum* that *nasST* genes regulate respiratory nitrous oxide and periplasmic nitrate reductases [41]. Therefore, additional targets for NasT may exist in *P. denitrificans*, which could function as negative regulators of *nar* gene expression.

In the *nasS* mutant, the *nasABGHC* gene expression is no longer under nitrate control because in the absence of the NasS protein, all the NasT pool is free to serve as a transcription antiterminator under all growth conditions (Figure 3B,C). Thus, in contrast with the wild-type strain, the *nasS* mutant of *P. denitrificans* displayed in 2D-PAGE gels the assimilatory nitrite reductase NasB and the Rieske-type NasG polypeptides when glutamate was used as the nitrogen source, independently of the presence or absence of nitrate (Figure 3C). These results corroborate that nitrate exerts a regulatory control at post-transcriptional (translational) level in addition to the role of NasTS in the transcription antitermination.

The phenotypes of the *ntrB*, *ntrY*, and *ntrB*/*ntrY* mutants of *P. denitrificans* suggest that the NtrBC system is mainly required for nitrate assimilation, while the NtrYX system has only a slight contribution (Figure 5A). The regulatory *nasTS* genes and the structural *nasA* gene are down-regulated in the *ntrB* mutant of *P. denitrificans* (Figure 5B), suggesting that these genes are under the control of the NtrBC system. This idea has been corroborated using the *lacZ* gene transcriptionally fused to native and mutated *nasT* leader sequence (Table 3). These results indicate that, in the absence of ammonium, phosphorylated NtrC could bind to the *nasT* promoter region, where a consensus NtrC-binding site has been identified. The absence of the *nasABGHC* transcript in cells grown with ammonium also suggests that NtrC or NrtX may bind to an as-yet-unidentified site in the *nasA* leader region. According to the RegPrecise program, very well-conserved putative NtrC-binding sites are found in the promoter regions of the *nasT* and *ntrBC* genes, suggesting that these two gene clusters are under the regulation of the general nitrogen control.

**Figure 6.**
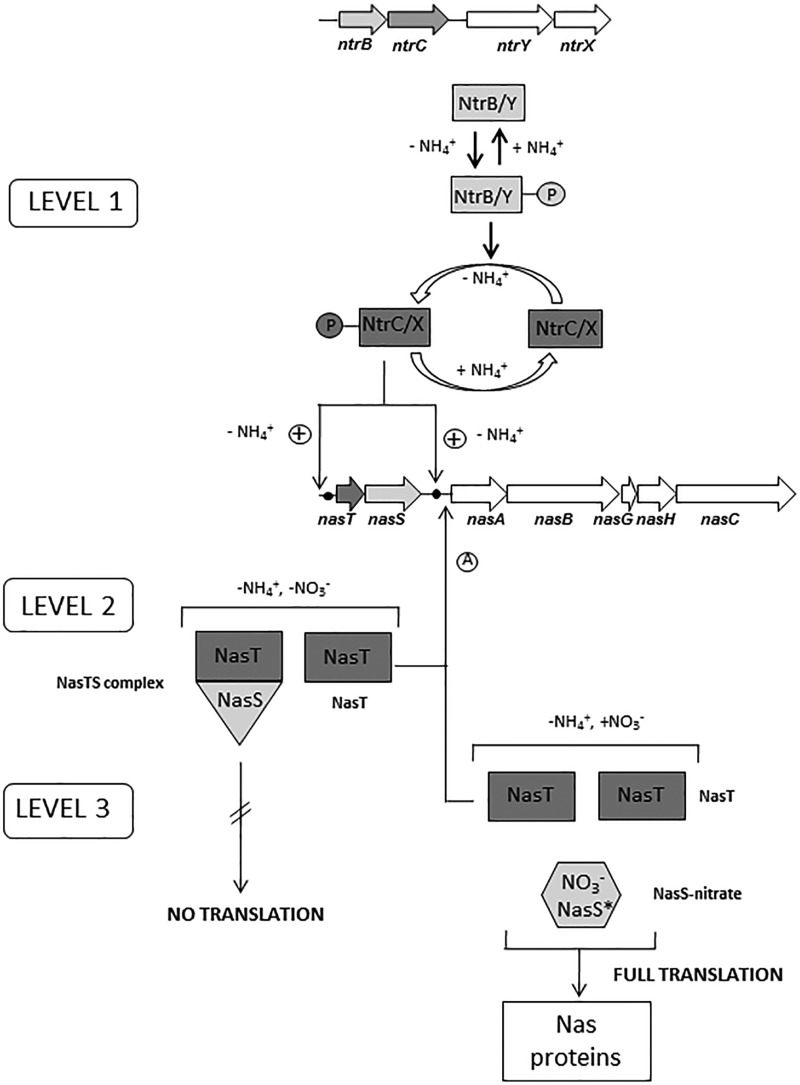
Proposed model of the nitrate assimilation regulation in *P. denitrificans*. The model describes the regulatory cascade controlling expression of nitrate assimilation *nas* genes by the *ntrBCYX* gene cluster in response to ammonium availability and by the *nasTS* genes for nitrate control. Levels 1 and 2 include regulatory mechanisms that control transcription of the *nas* genes, whereas level 3 involves a control on the translation of the Nas proteins. A circle with a plus symbol means activation of the transcription and a circle with the capital letter A indicates transcription antitermination.

The increased synthesis of *P. denitrificans nasABGHC* genes reflects a system-specific response to ammonium-limited growth in which nitrate is initially the sole available N-source [2]. This bacterium also uses nitrate as the terminal electron acceptor by a membrane-bound nitrate reductase [1]. This respiratory nitrate reductase (Nar) can substitute for NasC (assimilatory nitrate reductase) during anaerobic growth. The present study shows that transcription of *nasTS* occurs at a low level in the presence of ammonium, whereas transcription of *nasABGHC* genes is fully repressed in cells grown with ammonium (Figure 2). In *K. oxytoca*, *nasR* expression is sensitive to ammonium since it is induced by the NtrBC system when ammonium is absent [14]. In *Azotobacter vinelandii*, expression of *nasST* genes is also sensitive to ammonium, although it is not under the control of the Ntr system [17]. Nevertheless, the capacity for nitrate assimilation is lost in several *ntr* mutants [19]. Recently, it has been demonstrated that NtrBC and NasST co-regulate the *nasAB* genes required for nitrate/nitrite uptake and reduction in *Azotobacter vinelandii* [20]. Bioinformatic analysis has revealed that the two-component NasTS system is a regulatory mechanism for nitrate assimilation that is widely spread among bacteria. Particularly, NasT is mainly present in α-proteobacteria, such as Rhizobiales and Rhodobacterales, and β-proteobacteria such as Burkholderiales [5]. NasTS is involved in the regulation of the assimilatory nitrate/nitrite reductase genes in response to nitrate or nitrite in *Azotobacter vinelandii*, *Pseudomonas putida*, and *Rhodobacter capsulatus* [17,18,42]. However, in *Azotobacter vinelandii*, these genes are arranged in the *nasST* orientation, instead of the *nasTS* organization found in *P. denitrificans*, and they do not cluster together with the *nas* genes encoding the nitrate/nitrite uptake and reduction system [43]. The NasS protein is homologous to proteins which belong to the ATP-dependent nitrate transport systems found in cyanobacteria, although NasS lacks the signal peptide for periplasmic translocation. This is consistent with the role of NasS as a nitrate/nitrite sensor rather than acting as the periplasmic component of the transport system for these oxyanions. The NasT protein is a regulator with the ANTAR-binding domain characteristic of transcription antiterminators like the *K. oxytoca* NasR protein [4,14,16]. However, the *nasTS* genes are absent in the *nas* cluster of *Klebsiella*, in which they are functionally replaced by *nasR*, a gene mainly found in γ-proteobacteria. It has been proposed that NasR is both a nitrate/nitrite sensor and a transcription antiterminator. A hairpin structure in mRNA upstream of the *K. oxytoca nas* structural genes causes early termination of transcription [14]. It has been suggested that in the presence of nitrate or nitrite, NasR binds to the mRNA transcript preventing hairpin formation. Additional novelty on the regulatory mechanisms for nitrate assimilation was found in *P. denitrificans*, because repression of structural *nas* genes by ammonium could be directly exerted by ammonium itself rather than by glutamine or the glutamine/2-oxoglutarate ratio. Under low nitrogen conditions (absence of ammonium), the NtrB and NtrY proteins become active to phosphorylate their respective partners, NtrC and NtrX, which, in turn, become active for binding to promoter regions of the regulated genes. In *R. capsulatus*, the histidine kinases NtrB and NtrY can substitute for each other as phosphodonors towards the response regulator NtrC [29]. Also, a cross-talk between the NtrB/C and NrtY/X sensor/regulator pairs has been suggested in *Azospirillum brasilense* during nitrate-dependent growth [26]. However, NtrY protein is much larger than NtrB, suggesting that they may play different functions. The NtrBC system is widespread among microorganism and displays known functions, like the control of genes for glutamine synthetase, amino acid transport, nitrate and nitrite assimilation, nitrogen fixation, and also for the expression of other nitrogen regulation genes. Although the role of the NtrBC system seems to be more related to nitrogen metabolism, in *Azospirillum brasilense*, there is also a link between this two-component regulatory system and polyhydroxybutyrate production [44]. To summarize, Figure 6 represents the integration of mutational, transcriptomic, and proteomic data to establish a novel regulation model of the nitrate/nitrite assimilation process in *P. denitrificans*, in which nitrate exerts a transcriptional and translational control that has no precedents in the literature as far as we know. In this model, three conditions have been considered: (i) the presence of ammonium, in which the *nasABGHC* genes are repressed and there is a very low expression of the *nasTS* genes; (ii) the absence of both ammonium and nitrate (i.e. glutamate as the N-source), in which *nasABGHC* and *nasTS* genes are expressed, but Nas polypeptides and nitrate reductase activity are not detected; and (iii) the absence of ammonium and the presence of nitrate, in which *nasABGHC* and *nasTS* genes are expressed and Nas proteins are synthesized, leading to an active nitrate assimilation system. Our experimental data support the existence of a regulatory cascade with three levels of control. At level 1, the phosphorylated NtrC protein (and probably NtrX in a lesser extent) interacts with the *nas* promoter to activate *nas* gene expression in the absence of ammonium, as described in other bacteria (general nitrogen control). Accordingly, a conserved NtrC-binding sequence has been found in the promoter region of the *nasT* gene. At level 2, the NasTS system controls expression of the *nasABGHC* genes by transcription antitermination. In the absence of nitrate, an inactive NasTS complex is formed, but there is enough free NasT protein to allow transcription antitermination for the synthesis of a complete *nasABGHC* transcript, which is detectable in cells grown with glutamate. This NasT/NasS unbalance is probably a consequence of the high level of expression of the *nasT* gene compared with the *nasS* gene. At level 3, translation of the Nas proteins occurs only in the presence of nitrate. This regulation at the translational level may occur when NasS binds to nitrate and the NasTS complex dissociates, leading to an increase in free NasT protein that, in turn, may lead to up-regulation of systems involved in post-translational regulation.

This is a novel regulatory mechanism, but bioinformatic analyses may suggest that NasTS regulation is emerging in a wide number of bacteria, as previously reviewed [8]. As the *P. denitrificans* Nas system is very well characterized [2,5], this work may now lead to comparative studies in other bacteria, in which the NasTS system regulated nitrate assimilation and even other processes like nitrous oxide reduction, as previously described in *B. japonicum* [41].

## Abbreviations

2D-PAGE: two-dimensional polyacrylamide gel electrophoresis
ANTAR: Ami and NasR transcription antitermination regulator
Cy5-dCTP and Cy3-dCTP: deoxycytidine triphosphate containing either Cy5 or Cy3 dyes
dNTPs: deoxyribose-nucleoside triphosphates
EDTA: ethylenediaminetetraacetic acid
Km: kanamycin
LB: Luria–Bertani
NADH: nicotinamide adenine dinucleotide
Nas: nitrate assimilation system
NIT: N-terminal nitrate/nitrite-sensor domain
Ntr: nitrogen regulator
PCR: polymerase chain reaction
PMF: peptide mass fingerprinting
RT-PCR: reverse transcription polymerase chain reaction
RT-qPCR: quantitative reverse transcription polymerase chain reaction.
